# Epidemiology of *Brucella* infection in the human, livestock and wildlife interface in the Katavi-Rukwa ecosystem, Tanzania

**DOI:** 10.1186/s12917-015-0504-8

**Published:** 2015-08-08

**Authors:** Justine A. Assenga, Lucas E. Matemba, Shabani K. Muller, Joseph J. Malakalinga, Rudovick R. Kazwala

**Affiliations:** Department of Veterinary Medicine and Public Health, Sokoine University of Agriculture, P. O. Box 3021, Morogoro, Tanzania; National Institute for Medical Research, P. O. Box 9653, Dar Es Salaam, Tanzania

**Keywords:** *Brucella*, Ecosystem, Interface, Epidemiology, RBPT, c-ELISA, PCR

## Abstract

**Background:**

Brucellosis is a zoonosis of public health importance worldwide. In Tanzania, the disease is underreported due to insufficient awareness, inadequate diagnostic protocols, including lack of appropriate reagents for diagnosis. Livestock and wildlife are considered potential sources of infection to humans; however, the role played by these carriers in the epidemiology of the disease in the ecosystems in Tanzania is not fully understood. The objective of this study was to establish the prevalence of anti-*Brucella* antibodies in humans, wildlife and livestock; and molecular prevalence of *Brucella* spp in cattle and goats in the Katavi- Rukwa ecosystem.

**Results:**

Anti-*Brucella* antibodies were detected in humans at 0.6 % (95 % CI: 0.1, 2.1 %); cattle at 6.8 % (95 % CI: 5.4, 8.5 %), goats at 1.6 % (95 % CI: 0.4, 4.1 %) and buffaloes at 7.9 % (95 % CI: 1.7, 21.4 %). One of the two sampled lions tested positive. Cattle had a significantly higher prevalence of anti-*Brucella* antibodies as compared to goats (*P* < 0.05). A significantly higher seroprevalence was found in female than in male cattle and in adult than in young cattle (*P* < 0.05). There was an agreement of 95 and 89 % in cattle and goats, respectively, for the Rose Bengal plate Test (RBPT) and Competitive Enzyme Linked Immunosorbent Assay (c-ELISA) in detecting *Brucella* infection. Eight (3.5 %) out of 231 milk samples tested were positive for *Brucella* spp on Polymerase Chain Reaction (PCR), and *Brucella abortus* biovar 1 was detected in cattle milk. However, no *Brucella* spp were detected in goat milk.

**Conclusion:**

This study has shown the presence of anti- *Brucella* antibodies in humans, livestock, and wildlife in the Katavi- Rukwa ecosystem. Transmission of the infection between wildlife, livestock and humans is likely to continue due to increasing human activities in the human wildlife interface. This information is an important contribution to public health policy development in the human wildlife interface of the Katavi- Rukwa ecosystem.

**Electronic supplementary material:**

The online version of this article (doi:10.1186/s12917-015-0504-8) contains supplementary material, which is available to authorized users.

## Background

Brucellosis is a contagious bacterial zoonotic disease of public health importance worldwide. The disease affects domestic animals, wildlife and humans and is caused by *Brucella* organisms [1]. The disease affects the reproductive system of animals, leading to considerable productivity losses, such as reduced milk production, abortion, weak offsprings, weight loss, cull and condemnation of infected animals due to infertility, lameness and impediment for trade and export [2]. In humans, the symptoms are not specific and are easily confused with other fever causing diseases such as malaria, typhoid fever, rheumatic fever, and arthroses [3]. Furthermore, there is reduced work capacity due to illness of the affected people, and the government incurring costs on research and eradication programme and loss of financial investment [2]. The source of infection for humans are infected domestic animals, wild animals and their products [4]. The disease is an occupational risk for farmers, veterinary surgeons, and workers within the meat industry [4]. The sources of infection for animals include aborted materials, vaginal discharges, milk and semen from infected animals [4]. Transmission in wildlife occurs through spill over from domestic animals and wild species [5]. Contacts between wildlife, livestock and humans are common among pastoral and agro-pastoral farming communities in Tanzania. This interaction favours unhindered disease transmission between wildlife, livestock and humans [6].

Brucellosis is one of the most widespread zoonoses in the world and is endemic in most African countries [7–11]. The driving factors of the epidemiology of the disease in wildlife, livestock and humans in Sub Saharan Africa (SSA) is not well known and the available data are inadequate [8]. In Tanzania, the first outbreak of brucellosis was reported in Arusha in 1927 [12]. Previous surveys in Tanzania have demonstrated the occurrence of the disease in cattle in various production systems, regions and zones with individual animal level seroprevalence varying from 1 to 30 % [6, 12–24]. There has been no isolation of *Brucella* for more than 50 years ago and at that time *B. abortus* and *B. melitensis* were isolated from cattle and small ruminants respectively.

In humans, the occurrence of the disease has been reported in many areas including: Manyara, Lake Victoria zone, Western zone, Arusha, Tanga Municipality, Northern Tanzania and Morogoro region with seroprevalence varying from 0.7 to 20.5 % [25–30]. A serosurvey carried out in Serengeti ecosystem indicated that 24 and 17 % of buffaloes and wildebeests populations respectively are exposed to *Brucella* spp [6]. However, there is no previous report on brucellosis in humans, livestock or wildlife in the Katavi-Rukwa ecosystem where there is a comparative interaction of humans, livestock, and wild animals. The objective of this study therefore was to establish the prevalence of anti-*Brucella* antibodies in humans, livestock, and wildlife (buffaloes, zebra and lions). In addition, molecular prevalence of *Brucella* spp in cattle and goats in the Katavi-Rukwa ecosystem has been demonstrated.

## Results

### Serological results

Five out of 340 (1.5 %) humans tested were positive to Rose Bengal Plate Test (RBPT) and 2 (0.6 %) were found to be positive after screening by Buffered Acidified Plate Antigen Test (BAPA). The RBPT positive samples were further confirmed by the Rivanol Precipitation Test (Riv.T) in which 2 samples (0.6 %) were positive at a titer 1:200. Eighty eight (6.5 %) out of 1351 cattle and goats sera tested positive with RBPT. The RBPT positive sera were retested with c-ELISA and 79 (5.8 %) were found positive. Based on c-ELISA results, the overall seroprevalence in cattle and goats was 5.8 % (95%CI: 4.6, 7.2 %). The individual animal species seroprevalence was 6.8 % (95%CI: 5.4, 8.5 %) for cattle, and 1.6 % (95 % CI: 0.4, 4.1 %) for goats (Table 1). A significantly higher seroprevalence of 5.2 % was observed in cattle than in goats (95 % CI: 2.4–7.2, *χ*^2^ = 9.0, *P* = 0.003).

**Table 1:** Prevalence of *Brucella* antibodies in cattle and goats in Katavi-Rukwa ecosystem

Species	No tested	No of positive animals (Prevalence)				95 % CI
		RBPT		c-ELISA		
		No	% positive	No	% positive	
Cattle	1103	83	7.5	75	6.8	5.4, 8.5
Goats	248	5	2.0	4	1.6	0.4, 4.1
Total	1351	88	6.5	79	5.8	4.6, 7.2

**Fig. 1:**
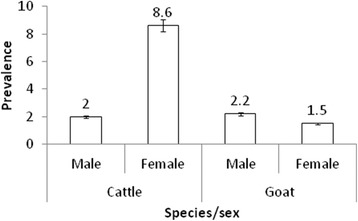
Sex related brucellosis seroprevalence in cattle and goats at Katavi-Rukwa ecosystem

**Fig. 2:**
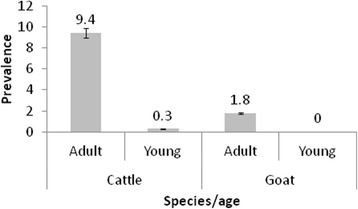
Age distribution in brucellosis seroprevalence in cattle and goats at Katavi-Rukwa ecosystem

The seroprevalence differed significantly between female and male cattle (difference 6.5 %, 95 % CI: 3.6–9.0, *χ*^2^ = 13.6) (*P* = 0.0002) (Fig. 1). However, there was no significant difference in seroprevalence between female and male goats (difference 0.7 %, 95%CI:-2.8–10.1, *χ*^2^ = 0.1) (*P* = 0.75). There was a statistically significant difference in seroprevalence between young and adult cattle (difference 9.1 %, 95%CI: 6.7–11.4, *χ*^2^ = 28.3) (*P* < 0.0001) (Fig. 2); but not in goats (difference 1.8 %, 95%CI: -15.1–4.4, *χ*^2^ = 0.1) (*P* = 0.741).

In buffaloes, 4 out of 38 sera (10.5 %) were positive by RBPT, while with BAPA 3 sera (7.9 %) were positive. Upon retesting the RBPT positive samples with Riv.T 3 (7.9 %) were found positive (95%CI: 1.7, 21.4 %) at a titer 1:200 (Table 2). The seroprevalence of *Brucella* infection was found to be 14.3 % (*n* = 14) in female, and 4.2 % (*n* = 24) in male adult buffaloes. In lions, the results were similar between RBPT, BAPA and Riv.T (one of the two lions tested was found positive at a titer 1:200). No *Brucella* antibodies were detected in zebra (*n* = 2). A comparative analysis between RBPT and cELISA is as presented in Tables 3 and 4.

**Table 2:** Prevalence of *Brucella* antibodies in humans, buffaloes, lion and zebra in Katavi-Rukwa ecosystem

Species	No tested	No of positive humans, buffaloes and a lion (Prevalence)							
		RBPT		BAPA		Riv.T		95 % CI	TITER
		No	% positive	No	%Positive	No	% positive		
Humans	340	5	1.5	2	0.6	2	0.6	0.1, 2.1	1:200^*^
Buffaloes	38	4	10.5	3	7.9	3	7.9	1.7, 21.4	1:200^*^
Lion	2	1	1/2	1	1/2	1	1/2		1:200^*^
Zebra	2	0	0	0		0	0		

*All positive samples were strong reactive by Rivanol Precipitation Test at a titer of 1:200

**Table 3:** Comparative analysis between Rose Bengal Plate Test and c-ELISA Results in cattle

c-ELISA results	Rose Bengal results				
	Positive (*n* = 83)	Negative (*n* = 1020)	Total	Apparent Prevalence	Test agreement
Positive (*n* = 75)	75	0	75	0.07	*k* = 0.95*
Negative (*n* = 1028)	8	1020	1028	0.93	
Total	83	1020	1103		
Apparent Prevalence	0.07	0.92			

*The agreement between RBPT and c-ELISA to detect *Brucella* infection was excellent (*k* = 0.95)

**Table 4:** Comparative analysis between Rose Bengal Plate Test and c-ELISA Results in goats

c-ELISA results	Rose Bengal results				
	Positive (*n* = 5)	Negative (*n* = 243)	Total	Apparent prevalence	Test agreement
Positive (*n* = 4)	4	0	4	0.02	*k* = 0.89^*^
Negative (*n* = 244)	1	243	244	0.98	
Total	5	243	248		
Apparent Prevalence	0.02	0.98			

*The agreement between RBPT and c-ELISA to detect *Brucella* infection was excellent (*k* = 0.89)

In lactating cattle and goats in which milk was sampled for *Brucella* DNA detection, 6.1 % (*n* = 231) sera were positive to c-ELISA. The specific seroprevalence of dairy cattle was 6.4 % (*n* = 218). All tested dairy goats (*n* = 13) were negative.

### Multiplex PCR results

**Fig. 3:**
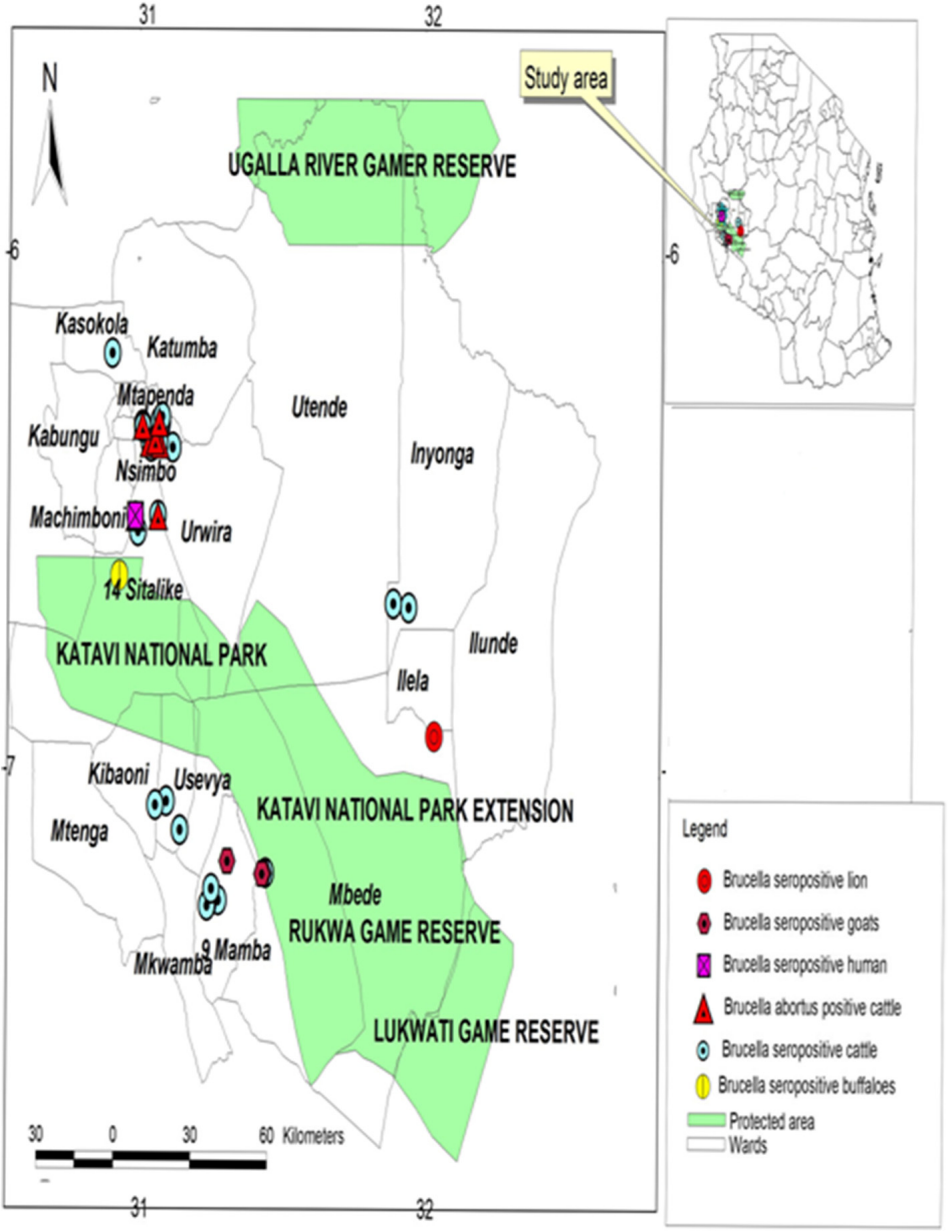
Map of Katavi region showing geographical distribution of *Brucella* antibodies in animals and humans and *Brucella abortus* biovar 1 in cattle milk

Eight out of 231 milk samples from cattle and goats tested positive with Multiplex PCR. Thus, the overall detection rate in cattle and goats was 3.5 %. The specific detection rate in cattle was 3.7 % (*n* = 218); while no *Brucella* species were detected from goat’s milk (*n* = 13). The *Brucella* species detected from cattle was *Brucella abortus* biovar 1. All PCR positive results were shown by migration of PCR product to approximately 495-bp for *Brucella abortus* fragments, with IS *711*-specific, *B. abortus*- specific and *B. melitensis*- specific primers indicating that it was *Brucella abortus* biovar 1 (Additional file 1). Geographical distribution of *Brucella* seropositive humans and animals, and *Brucella abortus* multiplex PCR positive cattle is as presented in Fig. 3.

## Discussion

The overall seroprevalence of *Brucella* spp in cattle and goats was 5.8 % based on the OIE recommended confirmatory c-ELISA test. The seroprevalence results agree with the results of earlier studies conducted in different parts of the country that show the prevalence as ranging from 1 to 30 % [14, 19, 22–27]. The infection suggests exposure to the bacteria because vaccination against brucellosis has not been carried out in Katavi region. Higher prevalence of the disease was demonstrated in cattle than in goats with significant variations (*P* < 0.05). Similar results were found in a study carried out in Mikumi-Selous ecosystem in Eastern Tanzania, in which cattle were the most infected with *Brucella* spp among different domestic ruminant [24]. The results are also similar to the findings in cattle, camels, and goats kept under pastoral management in Ethiopia [31]. Similar findings in ruminant species sharing a similar ecosystem in Chad have been reported [7]. In Nigeria the seroprevalence of the disease was higher in cattle than in goats [32]. Similar findings were revealed in a study conducted in Sudan [33]. The reason for this could be due to susceptibility to *B. abortus,* which was the only *Brucella spp* identified in this study. *Brucella abortus* infection is more prevalent in cows than in small ruminants in the study area. Furthermore, *B. melitensis* has been rarely reported in East African countries. Moreover, brucellosis studies carried out elsewhere in low income countries of Africa and Asia showed that most bovine brucellosis were caused by *Brucella abortus* although *B. melitensis* is becoming increasingly common [34].

This study shows further that *Brucella* seroprevalence in female cattle was significantly higher than in male cattle (*P* < 0.05). This finding is in agreement with the findings of studies previously conducted in Ethiopia, Nigeria and Pakistan [8, 35, 36]. The explanation is that females are kept for a comparatively longer period within the breeding herd than is the case with males and so increase their chance of exposure to infections [8]. Furthermore, the infection gets reactivated in females during pregnancy [37]. However, other studies have found no differences in brucellosis seroprevalence between male and female cattle [35].

In this study, *Brucella* seroprevalence was significantly higher in adult cattle than in young ones (*P* < 0.05). A similar observation has been reported previously [38]. This could be due to the fact that sex hormones and erythritol that stimulate multiplication of *Brucella* organisms, tend to increase in concentration with age and sexual maturity [8, 39].

In this study, the seroprevalence of *Brucella* infection in goats was 1.6 % which is significantly higher than that reported by a study conducted in Mikumi- Selous Ecosystem of 0.5 % [24]. Another study carried out in Manyara and Arusha regions of northern Tanzania showed a significantly higher seroprevalence of 6.0 % [27]. The difference may be due to the variation of risk factors from one geographical location to another, climatic factors and sampling techniques [27].

This study demonstrated a 0.6 % seroprevalence of *Brucella* antibodies in humans in Katavi-Rukwa ecosystem. This is lower than that reported previously [25, 26]. The reasons for the observed difference in these studies may be attributed to different in habit of the tribes of consumption of raw milk and milk products as well as contacts with the infected animals as explained previously [40]. Furthermore, the prevalence in Katavi region was lower than that found in Morogoro eastern Tanzania [30]. It is noted that the Morogoro study was carried out in hospital settings targeting people with febrile illness.

The study show further that *Brucella* antibodies are present in buffaloes at Katavi National Park with a seroprevalence of 7.9 %, which was lower than that recorded in Serengeti National Park of 24 % [6]. The difference might be due to the higher rate of animal migration into the Serengeti ecosystem as compared to Katavi National Park. In the Serengeti ecosystem, pasture and water are scarce during the dry season and wild herbivores migrate extensively to different areas such as the Maasai Mara in search of pasture and water. This migration increases contacts with cattle, and the chances of the wild animals picking up the infection. The difference can also be explained by the fact that the Serengeti ecosystem has a higher density of wild herbivores as compared to Katavi ecosystem resulting into elevated infection due to high frequency of animal contact [41]. The seroprevalence of *Brucella* infection in buffaloes showed comparable sex patterns of infection to livestock with females showing a higher seropositive as compared to males. However, the small number of positive cases limited significance testing in buffaloes. This finding was similar with that reported by other researchers [42]. *Brucella* antibodies were also detected in one of the two lions tested.

When c-ELISA results were compared with those in Rose Bengal Plate Test (RBPT), 79 samples were shown as positive in both tests. Although 88 samples were found positive with RBPT, only 79 samples were found positive with c-ELISA. Statistical analysis of the results demonstrated an excellent agreement between RBPT and c-ELISA test results because the test agreed 95 % of the time in cattle (Table 3) and 89 % in goats (Table 4). The c-ELISA confirmatory test “reduced” the number of positive animals from 88 to 79. This may be due to cELISA elimination of some reactions due to cross reacting bacteria. [2]. The RBPT is susceptible to cross reaction with other gram negative bacteria such as *Yersinia enterocolitica* O: 9, *E.coli* O: 157; and that some *Salmonella* spp, which could lead to false positive results as explained previously [1, 43]. Nonetheless, these two methods are suitable for the detection of *Brucella* antibodies in cattle and goats in Katavi-Rukwa ecosystem. Furthermore, the seropositive results were likely to be caused by field *Brucella* spp because the confirmatory test c-ELISA has high specificity, which minimizes false positives caused by cross-reacting antibodies of other gram negative bacteria or due to vaccination.

The RBPT has been validated in cattle and in small ruminants and revealed high sensitivity, hence its preference as the screening test in animal and human brucellosis [42, 44]. The c-ELISA has been validated in cattle with relatively high specificity; however it is less sensitive than RBPT. The test is an excellent confirmatory test for diagnosis of brucellosis in most mammalian species [45, 46]. Both tests are recommended by OIE as valuable livestock diagnostic tests [47]. The BAPA revealed the highest sensitivity and were used for screening whereas Riv.T showed highest specificity and were used as a confirmatory test in buffaloes. The main limitation of the study is that none of these tests have been validated in wildlife. Given the small sample size of two lions and zebra it is not clear if these animals have the infection or not.

In the present study *Brucella abortus* biovar 1 was detected in cow milk. This biovar has been detected in East African cattle and pose the risk of causing human infection especially in rural areas where milk and milk products are not pasteurized [48]. The colonization by *Brucella* spp of the mammary gland and its associated lymphnodes and consequently excretion of bacteria in milk has been previously described [49]. Accurate and fast evaluation of the status of the disease in milk and dairy products is essential for public health. The main limitation of this test is *B. abortus* biovars 3, 5, 6 and 9 are not detected by the test.

This study compared the serology and PCR findings of *B. abortus* in blood and milk samples collected from dairy cattle and goats. All the tested goats (*n* = 13) were negative for *B. abortus* on serology and PCR. The results indicated that 6.4 % of the sampled cattle (*n* = 218) were serologically positive and 3.7 % were PCR positive. All samples that tested positive on PCR also tested positive on serology. The proportion of seropositive cattle found to be PCR positive decreases with low level of active infection. This suggests that cattle exposed to *B. abortus* early in life may begin to recover from active infection after their first pregnancy following seroconversion. Similar findings were observed in bison [37]. The association of serological response with active infection in older cattle may indicate recent exposure of infection or recurrence of chronic infection [37].

This study detected *Brucella* spp antibodies in humans, cattle, goats, buffaloes and lions sharing the same ecosystem. Furthermore, the study detected *Brucella abortus* biovar 1 DNA from the cow milk. The transmission of the infection among these species in the ecosystem may be due to direct or indirect contact of infected and susceptible species through aerosol and contamination of feed. In the Katavi-Rukwa ecosystem, wildlife and livestock share grazing grounds as well as watering points. These animals inevitably contaminate their environment during calving with discharges which might be the source of infection to other animals while humans are often infected through occupational contact with the infected animals and their aborted materials and vaginal discharges. In the Katavi-Rukwa ecosystem, bush meat consumption is common and buffaloes are one of the main species hunted [50]. Apart from cattle, buffaloes may, therefore, be involved in the transmission of *Brucella* spp to humans in areas where buffalo meat is consumed.

## Conclusion

In conclusion, anti-*Brucella* antibodies were present in humans, cattle, goats, and buffaloes in Katavi-Rukwa ecosystem. This suggests that the infection might circulate within these species and maintained in the ecosystem. Furthermore, this study detected *Brucella abortus* biovar 1 in cow milk indicating that this species is released through milk and poses a public health risk to the milk consumers. Low seroprevalence in goats might be due to the fact that *B. melitensis* the aetiological agent in goats is relatively rare in the sub-Saharan region.

## Methods

### Description of the study area

This study was carried out in Katavi region, south-west of Tanzania. The region was purposively selected for the study because its districts border with a national park and game protection areas; and have large numbers of animals and human populations. The communities inhabiting the study area practice agro-pastoral farming system with a wide range of livestock, which is at times mix with wild animals in Katavi National Park. The region is located around 60 30′S and 31^0^ 30′E. The study was carried out between September 2012 to July 2013 covering three districts of Katavi region, which are Mpanda, Mlele and Nsimbo. The region has warm and cool dry season with considerable variation of rainfall from year to year. The area has a long rainy season which extend from November to April and a dry season which extends from May to October. The breeds of cattle reared are mainly the indigenous short horn zebu, ankole, boran with few cross breeds (indigenous and exotic). Indigenous sheep and goat breeds are also kept. The residents mainly cultivate food crops (maize, rice, sweet potatoes and beans) and cash crops (tobacco, groundnuts, simsim and sunflower). Despite that agro-pastoralists have permanent settlement, where they practice free range type of grazing during the rainy season. Animals graze on crop residues after harvest and thereafter, the animals are transferred to distant grazing lands known as “grazing camps” during the dry season. Transferring of animals normally begins in early August and animals are brought back in early November at the beginning of the rainy season. Katavi National Park comprises seasonally flooded grassland plains, miombo woodlands, small lakes and swampy wetlands [50]. Wild animals which are commonly found in the park include African buffaloes (S*yncerus caffer*), elephants (*Loxondata africana*), zebras (*Equus burchelli*), impalas (*Aepyceros melampus*), giraffes (*Giraffa camelopardalis*), eland (*Tourotragus oryx*), baboon (*Papio spp*), hippopotamus (*Hippopotamus amphibious*) and predators such as lion (*Panthera leo*), and other small carnivores [51].

### Study design and sample size estimation

A cross-sectional study was conducted to determine the epidemiology of *Brucella* infection in humans and animals in three districts making up the Katavi region. The sample sizes for humans and animals for serological studies, milk samples from cattle and goats for molecular studies, were calculated by the formula of multistage random sampling described by [52]. The human sample size was based on previous brucellosis prevalence studies in humans of 8.3 % in pastoral and agropastoral communities in Tanzania [27]. For cattle, sample size was based on brucellosis herd prevalence of 1–30 % in Tanzania [17, 25, 26] and it was assumed that 30 % of the cattle within the infected herd will have brucellosis. For goats, the sample size was based on brucellosis of individual animal prevalence of 0.5–6.0 % in Tanzania [24, 27] and it was assumed that 6 % of the goats within the infected herd will have brucellosis. For milk samples from cattle and goats for molecular studies, sample sizes were based on brucellosis herd prevalence of 5.3 and 5.3 % in dairy cattle and goats, respectively [53]. Based on these assumptions the sample size was estimated for expected prevalence (P_exp_) of 8.3 % (humans), 30 % (cattle), 6 % (goat), 5.3 % (milk samples) with 80 % power and 95 % confidence interval at 5 % desired precision were applied using the formula *n* = Z^2^*P*_exp_ (1 –*P*_exp_)/L^2^ [52], where Z is confidence level, L is desired precision and P_exp_ is expected prevalence. Furthermore, the confidence level of 1.96 was used. In this study design effect of 2.18 and rho of 0.09 were used as described previously by [54]. Therefore the design effect (D) of the survey was calculated using the formula *D* = 1 + (*b* – 1) *roh* [55], where *b* is the average number of samples per cluster and *roh* is the rate of homogeneity, equivalent to the intra-cluster correlation coefficient (*ρ*) in single-stage cluster sampling. The required sample size for humans was N*D which is 117* 2.18 = 256 was estimated. However, more human subjects (340) were sampled based on the interest of the communities i.e. we got more volunteers for blood sampling. From the sample size calculation 702 cattle (323 * 2.18 (Design effect)); and 190 goats (87* 2.18) were to be sampled for serological studies but due to cooperation with agro-pastoralist and field officers we were able to sample 1103 cattle and 248 goats which totals 1351 livestock from 36 villages. The estimated sample size for milk samples were 171 for cattle and 171 for goats, respectively. However, the total sample sizes for milk collected from cattle and goats were 218 and 13, respectively.

### Sampling procedure

A random sample of 36 villages was done, using a table of random numbers, from a sampling frame comprised of a list of all villages in the study area, which were made available by district livestock officers. Proportion sampling was adopted to obtain the number of villages from each of the three study districts. Within each village five households were randomly selected as the primary sampling units. The criterion for household inclusion in the sampling frame was any household composed of herders who keep cattle and/or goats for whatever purpose. The number of animals to be sampled from each herd was determined as described previously [52]. At herd level the animals to be sampled were randomly selected using a table of random digits. In this case eight (8) animals were sampled per household as suggested previously [52]. The ratio of goats to cattle sampled from a household keeping both goats and cattle was 1:4, based on the average ratio of goats to cattle in the study area. Adult animals were frequently sampled contributing 75 % as opposed to young animals which constituted 25 %, because *Brucella* infection is dependent on age. In this study, we defined adult cattle as the one aged two years and above; and a young one having less than two years. Adult goats, which were chosen are the ones aging one year and above; while a young one having less than one year. The cattle and goats were selected randomly for milk sampling using multistage random sampling formula as described above. Milk samples were taken from 231 lactating cattle and goats. For the selected households human subjects were also sampled regardless of their health status. A total of 42 wild animals were sampled opportunistically. The wild animals, including buffaloes were sampled from across the wildlife reserve.

### Sample collection

Following verbal consent, human blood samples were taken from the brachial veins using 5 ML plain vacutainer tubes. Cattle and goats were manually restrained and had blood samples taken from the jugular vein using 10 ML plain vacutainer tubes. Buffaloes were captured by darting using a combination of 5–8 Mg Etorphine hydrochloride (M99 9.8 Mg/ML) (Novatis, South Africa) and 50–80 Mg azaperone tartarate, while zebras were immobilized using a combination of 6–7 Mg M99 and 80 Mg azaperone. Lions were immobilized using a combination of 2.5 Mg/kg Ketamine hydrochloride and 0.1 Mg/kg medetomidine hydrochloride (Kyron, Pty, SA). The drug was remotely injected using a darting gun. The antidote Diprenorphine hydrochloride (M5050) (Novatis, South Africa) was used to revive buffaloes and zebras after collection of the blood. Lions were revived using antisedan (atipemazole hydrochloride) after the animal had stayed under anaesthesia for more than 60 min to give enough time for the body to metabolize much of the ketamine HCl. Blood from these animals was collected from jugular vein using 10 ML plain vacutainer tubes. The blood samples from humans, domestic and wild animals were allowed to clot in a slant position and serum samples were harvested after 24 h. The harvested serum was transferred to 1.5 ML cryovials and stored in Liquid Nitrogen (LN) before being transferred to the laboratory at the Faculty of Veterinary Medicine, Sokoine University of Agriculture where they were stored in an ultra deep freezer (−80 °C) until tested.

Cows and does from each selected households were randomly selected and sampled to obtain a total of 231 animals. Milk samples were collected under hygienic condition from udders of cattle and goats by hand stripping just prior to milking using sterile screw caped 50 ML falcon tubes. Each sample was composed of representative amount of milk taken from each quarter. Volumes of about 12 ML of milk sample were taken from each quarter to have a total of 50 ML of milk from cattle and 24 ML of milk from goats. First streaps of milk from each quarter were discarded. Blood was collected from lactating cattle and goats in which milk was sampled for *Brucella* DNA detection using 10 ML plain vacutainer tubes for serological study. The samples were immediately stored in LN before transferred to the laboratory at the Faculty of Veterinary Medicine, Sokoine University of Agriculture to be stored in ultra deep freezer (−80 °C) until tested.

### Serological analysis

Brucellosis testing in cattle, goats, humans, buffaloes, zebra and lions was based on a panel of serological diagnostic tests. The Rose Bengal Plate Test (RBPT) and the Buffered Acidified Plate Antigen Test BAPA were used as screening tests; cELISA and Riv.T were used as confirmatory test. The RBPT is used for detection of *Brucella* antibodies in livestock, wildlife and humans and has been validated in cattle with 98.1 % sensitivity and 99.8 % specificity, and acceptance accuracy for diagnosis in African buffalo (98.6 % sensitivity and 99.2 % specificity) [42]. The BAPA (sensitivity 97.4 % and specificity 60 %) has been validated in domestic ruminants [44] while cELISA may be used to test samples from different species simultaneously and has high sensitivity (95.2–99.4 %) and specificity (98.9–99.7 %) [45, 46]. The test has been validated in cattle [56]. The Rivanol Precipitation Test (Riv.T) has been validated in cattle and small ruminants (sensitivity 85 % and specificity 87.6–100 %) [44, 56].

The RBPT (Central Veterinary Laboratory, New Haw, Addelestone Surrey KT153NB, UK) was used for screening *Brucella* antibodies in cattle and goats as described previously [57]. The results were confirmed by c-ELISA diagnostic kit as per the protocol described by Animal Health and Veterinary Laboratory Agency (AHVLA) New Haw, Addelestone Surrey KT153NB, United Kingdom; according to the recommendations from the World Health Organization for animal health [1]. The optical density (OD) was measured at 450 nm using ELISA reader Multiscan RC Version 6.0 (Thermo labsystem, Helsinki, Finland). The positive/negative cut off was calculated as 60 % of the mean OD of four conjugate control wells. A test sample giving an OD equal to or below this value was regarded as positive. Validation of cELISA test was carried out with positive and negative controls as per the manufacturer’s instruction. All samples were tested in duplicate and where the plate validation failed the procedure was repeated. A sample was considered positive if was positive for both RBPT and cELISA.

In humans and wildlife, sera were double screened by RBPT and BAPA (National Veterinary Service Laboratory, Ames, lowa, USA). The results were confirmed by Rivanol Precipitation Test as described by National Veterinary Service Laboratory, Ames, lowa, USA, according to the method described previously [58, 59], where the reactive samples from RBPT or BAPA were serially diluted at 1:25, 1:50, 1:100 and 1:200 using Standard Plate Test (SPT) solution to establish the titer of the reactive sera.

Rose Bengal Plate Test (Central Veterinary Laboratory, UK), was used for screening sera from lactating cattle and goats in which milk was sampled for *Brucella* DNA detection as described previously, [57]; and the results were confirmed by c-ELISA as per the protocol described by AHVLA U.K.

### Extraction of DNA from milk samples for PCR assay

Volumes of 15 ML of frozen milk sample was thawed and centrifuged at 14,000 G for 10mn. The supernatant, including whey portion, was separated and discarded and the sediment was subjected to genomic DNA extraction. The enzymatic lysis buffer was prepared composing of 20 mM Tris.Cl, pH 8, 2 mM sodium EDTA, 1.2%Triton^®^ X-100; and immediately before use, add lysozyme to 20 Mg/ml. DNA extraction of *Brucella* was carried out using genomic DNA purification kit (PureLink™ Invitrogen, Carlsbad, CA), as per the manufacturer’s instructions.

### Conventional PCR technique

The PCR assays which amplified the IS711 genetic element regions of the *Brucella* genome [60] were used with slight modifications. PCR reaction was made in 20 μl of reaction mixture as per mastermix instruction containing 10 μl Amplitag Gold^®^ Fast PCR Master mix (Applied Biosystems, Foster city, USA), 0.5 μl from each of *B. abortus* -specific primer, *B. melitensis* -specific primer and *IS711*- specific primer, 1 μl extracted DNA and 7.5 μl of nuclease free water to complete the mixture of 20 μl reaction. The reaction mixture was dissolved by vortexing and centrifuged briefly. The oligonucleotide primers used in this study were 5′- TGC-CGA-TCA-CTT-AAG-GGC-CTT-CAT-TGC-3′(IS711-specific primer), 5′-GAC-GAA-CGG-AAT-TTT-TCC-AAT-CCC-3′(*B. abortus*- specific primer) and5′- AAA-TCG-CGT-CCT-TGC-TGG-TCT-GA-3′ (*B. melitensis*- specific primer)(Additional file Additional file 2). The PCR reaction was performed in a Takara PCR Thermal Cycler (Takara Bio Inc. Japan). The condition for amplification of *Brucella* spp was initial denaturation at temperature of 95 °C for 10mn, followed by 40 cycles consisting of 95 °C for 30s, 55 °C for 30s, 72 °C for one minute and the final extension was at 72 °C for 10mn. A DNA lader with 50–2000-bp was used as a molecular weight maker. The amplified products were analysed by electrophoresis through a 1.5 % agarosegel (Sigma Co., USA) for 1 h at 100 Volts (V) with 0.5 × TBE buffer (89 mM Tris-HCl, 89 mM boric acid, 2 mM EDTA, pH 8). Gels were stained with Gelred (Phenix Research Products, Candler, NC28715, United States) and bands were detected with a UV transilluminator, and photographed. Visible band appropriate size of 495 bp for *B. abortus* and 730 bp for *B. melitensis* were considered positive. RB51 was used as positive control for *Brucella abortus* and REV.1 for *Brucella melitensis* while master mix without DNA template was used as negative control. Test validation was performed with two vaccine strains notably *Brucella abortus* RB51 and *B. melitensis* REV.1 as positive controls. DNA was extracted from RB51/ REV.1 and assay was performed as described above using PCR reaction. The PCR product was detected as described above. The test was repeated whenever the validation fails. The main limitation of this study is *B. abortus* biovars 3, 5, 6 and 9 are not detected by the test.

### Data analysis

Microsoft Office Excel^®^ 2007 (Microsoft Corporation, One Microsoft Way, Redmond, 98052-7329, USA) was used in storing data and drawing graphs. Data was analysed using Epi-Info version 7 (CDC Atlanta, USA) and MedCalc^®^ version 13.0.2 (MedCalc software, Acacialaan 22, B-8400, Ostend, Belgium). Chi square test was performed to calculate P value for the incidence rate of *Brucella* versus the different age groups, sex and species. P value < 0.05 was considered statistically significant.

### Ethics statements

The ethical clearance for conducting this study was granted by the Institutional Review Board of Sokoine University of Agriculture (SUA/FVM/R.1/9) and Medical Research Coordinating Committee of the National Institute for Medical Research, reference number NIMR/HQ/R8a/Vol.IX/1627. Permission to conduct the study in the National Parks and protected areas was sought and granted by Tanzania Wildlife Research Institute (TAWIRI) and the Officer in Charge of the Tanzania National Park (TANAPA). Permission to conduct the study in Katavi region; and Mpanda, Mlele and Nsimbo districts authorities was sought and granted by regional and district livestock officers for animal studies and regional and district medical officers for human studies respectively. Additionally, the verbal consent was sought from each participant after the author had explained the aim of the study to them. Verbal rather than written consent was sought since a number of agro-pastoralists in the study area are not literate. This study adhered to Tanzania Animal Welfare Act [61] and to the guidelines adapted to the Australian government [62].

## Additional files

Additional file 1:
**PCR products amplified from** ***Brucella abortus*** **DNA extracted from cattle and goats milk.** Lane M and 10 are 50–2000 bp molecular weight marker, Lane 1 Positive control *B. abortus*, lane 2: Positive control *B. melitensis*, Lane 3 is negative control, lane 4–9 are representative *B. abortus* biovar 1positive samples. (TIFF 102 kb) Additional file 2:
**Primers used in this study.** (DOC 26 kb)

## Abbreviations

c-ELISA: Competitive Enzyme Linked Immuosorbent Assay
RBPT: Rose Bengal Plate Test
Riv.T: Rivanol precipitation test
BAPA: Buffered acidified plate antigen test
CI: Confidence interval
PCR: Polymerase chain reaction
RB51: *Brucella abortus* rough strain 51
Rev.1: *Brucella melitensis* biovar 1 Rev 1
OD: Optical density
