# Supplementary figures and images for: Epidemiology of Brucella infection in the human, livestock and wildlife interface in the Katavi-Rukwa ecosystem, Tanzania
Source: BMC Vet Res. 2015 Aug 8;11:189. doi: 10.1186/s12917-015-0504-8 (PMC4529704; doi:10.1186/s12917-015-0504-8)

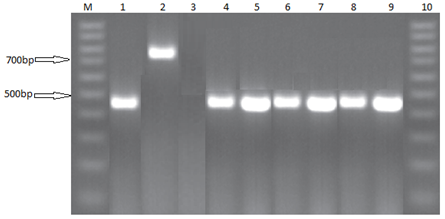

Supplement: Additional file 1: — PCR products amplified from Brucella abortus DNA extracted from cattle and goats milk. Lane M and 10 are 50–2000 bp molecular weight marker, Lane 1 Positive control B. abortus, lane 2: Positive control B. melitensis, Lane 3 is negative control, lane 4–9 are representative B. abortus biovar 1positive samples. (TIFF 102 kb) [file 12917_2015_504_MOESM1_ESM.tiff]
