# Supplementary material for: Epidemiology of Brucella infection in the human, livestock and wildlife interface in the Katavi-Rukwa ecosystem, Tanzania
Source: BMC Vet Res. 2015 Aug 8;11:189. doi: 10.1186/s12917-015-0504-8 (PMC4529704; doi:10.1186/s12917-015-0504-8)
Supplement: Additional file 2: — Primers used in this study. (DOC 26 kb) [file 12917_2015_504_MOESM2_ESM.doc]

**S2 Table**: Primers used in this study

| Primer | Nucleotide sequence 5’-3’ |
| --- | --- |
| IS 711-specific | TGC-CGA-TCA-CTT-AAG-GGC-CTT-CAT-TGC |
| *B. abortus* -specific | GAC-GAA-CGG-AAT-TTT-TCC-AAT-CCC |
| *B. melitensis* -specific | AAA-TCG-CGT-CCT-TGC-TGG-TCT-GA |
